# Statistics of Suicides in Paris

**Published:** 1847-10

**Authors:** 


*Statistics of Suicides in Paris.*—By a criminal report, for the year 1845, just published, out of 11,049 deaths, there were 3084 cases of suicide,—being 111 above above the number for 1844, and only 64 above that of 1843. Of the 3084 suicides, there were 2332 males and 725 females. Sixteen males and four females had not reached their sixteenth year. Among the number were children of 7, 8, and 10 years. There were—

| | |
|---|---|
| From 16 to 21 years - - - - | 123 |
| From 21 to 30 " - - - - | 362 |
| From 30 to 50 " - - - - | 1201 |
| From 50 to 70 " - - - - | 945 |
| From 70 to 80 " - - - - | 203 |
| More than 80 " - - - - | 41 |

Taking the months of the year, there were of suicides in

| | |
|---|---|
| Summer.—June July, and August - | 922 |
| Spring.—March, April, and May - | 861 |
| Autumn.—Sept., Oct., and Nov. - | 756 |
| Winter.—Dec., Jan., and February - | 545 |

According to the means of perpetration, there were—

| | |
|---|---|
| By hanging - - - - - | 1110 |
| " drowning - - - - - | 995 |
| " fire-arms - - - - - | 432 |
| " suffocation by charcoal vapour - | 213 |

The last mode of self-destruction is exceedingly prevalent in the department of the Seine.

The motives were those usually met with—love, jealousy, debauchery, reverse of fortune, domestic misery, and physical suffering. —*Ibid, from Ibid.*

